# Pure-Food Legislation

**Published:** 1899-12

**Authors:** 


### Pure-Food Legislation.

Unless the matter be forcibly brought to our attention it is not easy to realize how generally we are imposed upon by those whom we may properly call the manufacturers of food. At a recent meeting in Boston of the Farmers' National Congress, Hon. H. C. Adams, Dairy and Food Commissioner of Madison, Wis., presented facts which are well worth our serious consideration. From his showing it would appear that practically all of our food staples may be and often are adulterated, or actually made outright from substances far removed from the advertised finished product. No doubt the possibility of defrauding the public in this matter increases year by year, as methods of chemical analysis and synthesis are coming to be more generally recognized and understood. It is an easy and a tempting step from such knowledge to its fraudulent use in the manufacture of products which are foisted on the unsuspecting buyers to their detriment and to the great gain of manufacturers. Some of the possibilities in this line we quote from Mr. Adams' remarks:

"The clumsy wooden nutmeg of Connecticut, that even a policeman might detect, has given way to artificial eggs which no hen would recognize, and to artificial butter that never knew milk. The universal demand for cheap things brings a supply. Wheat flour is adulterated with corn flour, buckwheat with wheat middlings. Vermont maple syrup is made that never saw Vermont, and is made from the sap of trees that grow in Chicago. Glucose has dethroned cane syrup. Cider vinegar is distilled from grain. A good portion of the strained honey of commerce never produced any strain upon the bees. Milk is robbed of its cream, filled with lard and sent all over the world to ruin the reputation of American cheese. Oysters are partially embalmed with chemicals. Lemon extracts are made without lemon oil, and vanilla extracts without vanilla. The hogs of the North compete with cheap cotton-seed oil of the South and mix in the same tub under the banner of lard. Artificial smoke is made for hams out of poisonous drugs. Jellies colored in imitation of the natural fruits and sold as fruit jellies flood the markets, although they are almost as destitute of fruit juice as a bar of pig iron. The embalmed beef business has been exaggerated, but we do not need any for either soldiers or civilians. Canned fruits are preserved with antiseptics which delay the digestive processes. Baking powders under various misleading names crowd the markets. Spices enriched with pepper hulls and ground cocoanut shells are manufactured and sold by the ton. The close partnership which has existed for so many years between coffee and chickory does a thriving business in many States under the firm name of coffee. Cheapness is secured by these adulterations and false labelings, but the people are defrauded."

We quote the above statement as an example of what is going on about us, from which the poor, who need legislative protection most, are the chief sufferers. The manufacture of oleomargarine as a substitute for butter is a case in point. The necessity for legislation as applied to this product is said to be recognized now by thirty-two States, which have prohibited its sale under the guise of butter. Massachusetts has been a pioneer in the movement for improved laws, and experience has shown that State laws are usually sustained by the courts. Mr. Adams feels very strongly that we need a comprehensive national pure-food law which shall so regulate the general question of food that its adulteration for the benefit of the few will no longer be possible. It is evident that such legislation must be somewhat slow in coming; that it will be bitterly opposed and vigorously contested in the courts by the manufacturers of adulterated foods. In the meantime, however, it is desirable that our attention should be called to the matter, and that both as individuals and as a profession we should do what lies in our power to combat what is clearly a growing evil of very great magnitude.—*Boston Med. and Surg. Journal.*

